# Preparation, Characterization, and Photocatalytic Performance of Ag/BiOBr_0.85_I_0.15_ Nanocomposites

**DOI:** 10.3390/ma15176022

**Published:** 2022-08-31

**Authors:** Xiaobin Hu, Mingxing Zhao, Weihong Zheng, Junjie Zhu

**Affiliations:** School of Life Science, Huzhou University, No. 759, East 2nd Ring Road, Huzhou 313000, China

**Keywords:** Ag/BiOBr_0.85_I_0.15_, silver loading, photocatalysis, ammonia nitrogen degradation

## Abstract

In the present paper, a series of Ag/BiOBr_0.85_I_0.15_ composite nanoparticles with different silver loading were prepared by a combined solvothermal and photocatalytic reduction method. The composite samples have been characterized by XRD, XPS, SEM, EDX, TEM, UV-Vis, and N_2_ adsorption/desorption techniques. The characterization results showed that BiOBr_0.85_I_0.15_ composite nanoparticles have a tetragonal phase structure. Silver nanoparticles are uniformly distributed on the BiOBr_0.85_I_0.15_, which results in surface plasmon resonance absorption, effectively increasing the visible light absorption ability of BiOBr_0.85_I_0.15_. The photocatalytic activity of the samples was evaluated by photocatalytic degradation of ammonia nitrogen in circulating aquaculture water under simulated sunlight irradiation. The effect of the Ag loading amount on the photocatalytic degradation of ammonia nitrogen was investigated. Silver loading of 1% (molar ratio) can effectively improve the degradation capacity of the catalyst for ammonia nitrogen in water. The recycling experiments show that 1%Ag/BiOBr_0.85_I_0.15_ has good photocatalytic stability. ESR characterization and oxidation species scavenging experimental results suggest that h^+^, ^1^O_2_, and ·O_2^−^_ are the main oxidizing species in the photocatalytic system.

## 1. Introduction

Advanced oxidation processes (AOPs) have been widely studied and applied to organic wastewater treatment because of their high capability to oxidize and remove organic compounds and pathogens originated from agricultural, industrial, and domestic activities [1,2,3,4]. Photocatalysis technology is one of the most effective AOPs for pollutant degradation [5,6]. Under the excitation of light, the electrons of the semiconductor transit from the valence band to the conduction band, thus forming photogenerated electrons in the conduction band and photogenerated holes in the valence band. The oxidative photogenerated holes and reductive electrons can directly react with pollutants on the surface of the semiconductor, leading to their degradation. Moreover, photocatalysis reaction can produce reactive oxygen species (ROS), which play a key role in the oxidative degradation of organic compounds due to their high reactivity [5,6,7,8].

So far, many semiconductors have been studied as photocatalysts, such as TiO_2_ [9,10,11], ZnO [12,13], CdS [14,15], SrTiO_3_ [16], and so forth. Much attention has been paid to improving the photocatalytic ability of semiconductors. However, since the conventional photocatalysts with wide band gaps can only be excited under UV-light irradiation, their practical application has been greatly limited. Because ultraviolet energy accounts for only 4% of solar energy, visible light-driven photocatalysts attract more attention [17]. Therefore, visible-light-responsive photocatalysts have also been prepared, and proved to be effective in the degradation of organic dyes and toxic and bio-refractory organic compounds in water, such as g-C_3_N_4_ [18,19], WO_3_ [20], Ag_3_PO_4_ [21,22], Ag_3_VO_4_ [23,24], etc. In recent years, Bi-based compounds have been found to have high visible light photocatalytic activity, such as BiVO_4_ [25,26], Bi_2_O_3_ [27], Bi_2_Ti_2_O_7_ [28], Bi_2_WO_6_ [29], BiOBr [30], BiOI [31], AgBiO_3_ [32], etc. They possess narrow band gaps for their hybridized O 2p and Bi 6s valence bands, which makes them have high visible light catalytic activity [33].

Some solid solutions have been constructed as photocatalysts, showing good catalytic ability in the degradation of pollutants under visible light irradiation. Xu et al. [34] synthesized Zn_x_Cd_1−x_S solid solution photocatalysts which were extremely active for methylene blue photodegradation under visible light illumination. Zhang et al. [35] synthesized two-dimensional BiOCl_x_Br_1−x_ solid solution with exposed {001} facets and tunable band gaps by using solvothermal methods. The BiOCl_0.5_Br_0.5_ sample exhibited the highest photocatalytic activity for degrading rhodamine B. Wang et al. [36] developed a BiOBr_x_I_1−x_ solid solution to degrade 4-chlorophenol under visible light and found that the BiOBr_0.85_I_0.15_ sample showed very good catalytic activity.

On the other hand, much research work has focused on preventing the rapid recombination of photogenerated electrons and holes through a variety of methods to improve the photocatalytic degradation efficiency, such as semiconductor composite [37,38], ion doping [39,40], noble metal deposition [41,42], and so on. Noble metals on the surface of semiconductors can form a Schottky barrier at the contact interface between noble metals and semiconductors, inhibit the recombination of photogenerated hole–electron pairs, and prolong the lifetime of photogenerated carriers.

As the content of ammonia nitrogen (NH_4_^+^-N) is a very important water quality index that affects the aquatic environment of aquaculture, the degradation of ammonia nitrogen is very important. On the other hand, the aquaculture water system contains a variety of nitrogen-containing organic compounds from fish feed residues, fish excreta, and secretions, which may produce ammonia after a series of transformations. In recent years, there have been studies on the direct removal of NH_4_^+^-N through a photocatalytic reaction. Under the action of oxygen-containing free radicals, ammonia can gradually lose hydrogen atoms and finally convert to N_2_ [43]. In this research, a series of Ag/BiOBr_0.85_I_0.15_ composite nanoparticles with different silver loading were synthesized via the solvothermal method and the photocatalytic reduction method. The composition, structure, morphology, and optical properties of the photocatalysts were characterized. The photocatalytic activity of the catalysts was investigated by the degradation of NH_4_^+^-N in aquaculture wastewater under simulated sunlight irradiation. The photocatalytic mechanism of the photocatalyst was also discussed.

## 2. Preparation and Experimental Details

### 2.1. Photocatalyst Preparation

The solvothermal method was used to synthesize the BiOBr_0.85_I_0.15_ nanoparticles [36]. In a typical process, 1.215 g bismuth nitrate pentahydrate (Bi(NO_3_)_3_·5H_2_O) (Sinopharm Chemical Reagent, Shanghai, China) and 1.000 g polyvinylpyrrolidone (PVP, K-30) (Zhanyun Chemical, Shanghai, China) were mixed with 60 mL glycerol (Zhanyun Chemical, Shanghai, China) and 60 mL H_2_O. The mixture was stirred to form solution A. Then, 0.219 g sodium bromide (NaBr) (Zhanyun Chemical, Shanghai, China) and 0.0622 g potassium iodide (KI) (Zhanyun Chemical, Shanghai, China) was dissolved into another 20 mL H_2_O under magnetic stirring to form solution B. Subsequently, under constant agitation, solution B was added to solution A drop by drop in 1 h at room temperature. The suspension was transferred into 50 mL Teflon-lined stainless-steel autoclaves for hydrothermal treatment, and kept at 150 °C for 8 h, and then cooled down to room temperature naturally. After filtration, the product was washed with deionized water and absolute ethanol three times, respectively, to remove other impurities, and then dried at 80 °C for 12 h.

Ag nanoparticles were deposited on the surface of BiOBr_0.85_I_0.15_ by a photo-reduction approach [44]. The composite photocatalyst, 1.0% Ag/BiOBr_0.85_I_0.15_ (molar ratio of Ag to BiOBr_0.85_I_0.15_ = 0.01:1), was prepared as follows: 0.0054 g AgNO_3_ was dissolved in 50 mL methanol under stirring. Then, 1.000 g of BiOBr_0.85_I_0.15_ nanoparticles were added into the methanol solution while vigorously stirring to form a suspension. The suspension was irradiated with an ultraviolet lamp (λ = 365 nm, 30 w) for 6.0 min. The radiation intensity received at the reaction liquid surface was 12.38 W/m^2^ (CAS 140CT Array Spectrometer, Instrument Systems, Munich, Germany). The photocatalytic reduction of silver ions enables the synthesis of Ag/BiOBr_0.85_I_0.15_. Then, the suspension was filtrated and washed with deionized water and absolute ethanol, respectively. Finally, the product was dried at 80 °C for 2.0 h in an electric vacuum drying oven.

### 2.2. Characterization and Analysis

A transmission electron microscope, Talos F200X TEM (Thermo Scientific, Waltham, MA, USA), was used with about 0.005 g of the sample to obtain the morphology of the photocatalyst. A scanning electron microscope, ZEISS SUPRA 40 SEM (Carl Zeiss Microscopy GmbH, Oberkochen, Germany), was used with about 0.01 g sample to obtain the surface microstructure images of the catalysts, and energy-dispersive X-ray spectroscopy (EDAX) elemental mapping was conducted using a Bruker XFlash 6|10 detector (Bruker, Karlsruhe, Germany). X-ray diffraction (XRD) of the nanoparticles was measured by a D8 Advance X-ray diffractometer (Bruker, Germany, λ = 1.5418 Å, Cu Kα radiation) with a scanning speed of 5° min^−1^, using about 0.2 g of the sample. X-ray photo-electron spectroscopy (XPS) (ESCALAB 250Xi, Thermo Fisher Scientific, USA) with monochromatic Al Kα X-ray radiation at 1486.71 eV was applied to identify the surface element composition and chemical states of the as-prepared samples of the nanoparticles, using about 0.03 g of the sample. The specific surface area of the catalysts was determined by an ASAP 2020 analyzer (Micromeritics Instrument Corporation, Norcross, GA, USA) with an analysis bath temperature of 77 K, using about 1 g of the sample. Electron spin resonance (ESR) spectroscopy (Bruker EMX 10/12 spectrometer, Karlsruhe, Germany) was used to investigate the generation of reactive oxygen radicals in the photoreaction system. Hydroxyl radical and superoxide anion radical were captured by 5,5-dimethyl-1-pyrroline-N-oxide (DMPO), and singlet oxygen was captured by 2,2,6,6-tetramethylpiperidine (TEMP). The UV-vis diffuse reflectance spectra (DRS) of samples were obtained by a spectrophotometer (UV270, Shimadzu, Kyoto, Japan), using the BaSO_4_ as the reflectance sample with about 0.02 g of the sample.

The NH_4_^+^-N in water samples was analyzed using chromogenic spectrophotometry by a DR2800 multi parameter water quality analyzer (HACH Company, Loveland, CO, USA), following the HACH Water Analysis Handbook (HACH, Beijing, China, 2010). The NH_4_^+^-N was determined by the salicylic acid method.

The transient photocurrents of the as-prepared samples were investigated on an Electrochemical Working Station (CHI660E, Chenhua Instruments Co., Shanghai, China) in a standard three-electrode electrochemical quartz cell with Na_2_SO4 (0.5 M) electrolyte solution. A 500 W Xe light with a filter (λ > 400 nm) was employed as the light source. FTO glass with coated photocatalysts was used as the working electrode. A Pt foil and an Ag/AgCl electrode served as the counter and reference electrode, respectively. The working electrodes were prepared by the spin-coating method. The 10-mg powder sample was dispersed in 1 mL of absolute ethanol, and then 50 μL of Nafion ethanol solution was added, and the uniform suspension was formed by ultrasonic treatment for 30 min. Then, 150 μL of the suspension was added to the ITO glass and dried at room temperature to form a working electrode. The changes of the photoinduced current intensity with time were measured by controlling the cycle light on and off (turn on the light for 20 s, and then turn off the light for another 20 s).

### 2.3. Photocatalytic Experiments

A certain amount (for example, 0.08 g) of photocatalyst was accurately weighed and added to a beaker containing 100 mL of the water sample from a freshwater-recirculating aquaculture system. The water sample was filtered by an 0.22-μm membrane before photocatalytic degradation. The main water quality parameters of the filtered water sample are pH, 8.0; chemical oxygen demand (COD), 88 mg/L; total organic carbon (TOC), 55 mg/L; and ammonia nitrogen (NH_4_^+^-N), 50 mg/L. The catalysts in the water sample were dispersed by ultrasonic wave for 2.0 min. Subsequently, the suspension was stirred for 60 min in the dark to reach an adsorption–desorption equilibrium on the surface of the catalyst. The photocatalytic degradation of the water sample started under the irradiation of a 300 W xenon lamp. The distance between the light source and the liquid level was 15 cm. The radiation intensity received at the liquid surface of the degradation reaction was 7.416 W/m^2^ (CAS 140CT Array Spectrometer, Instrument Systems, Munich, Germany). The temperature of the degradation system was controlled by a cold trap using constant temperature cooling water. Then, 2.0 mL reaction suspension was taken out at certain intervals. All aliquots were filtered through a 0.22-μm filter membrane before analysis.

## 3. Results and Discussion

### 3.1. X-ray Diffraction (XRD)

**Figure 1:**
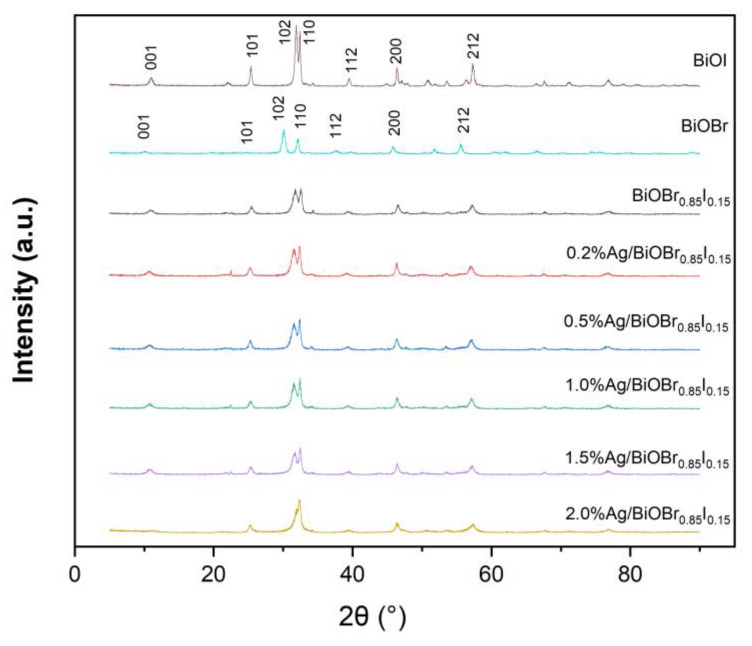
XRD patterns of BiOI, BiOBr, BiOBr_0.85_I_0.15_, and Ag/BiOBr_0.85_I_0.15_ with different Ag loading.

Figure 1 shows the XRD patterns of BiOBr, BiOI, and Ag/BiOBr_0.85_I_0.15_ composite catalysts with different silver contents. The diffraction peaks of BiOBr at 2θ = 11.04°, 25.38°, 31.92°, 32.40°, 39.56°, 46.40°, and 57.30° correspond to {001}, {101}, {102}, {110}, {112}, {200}, and {212} crystal planes of the BiOBr crystal, respectively. The position of the BiOBr diffraction peak is consistent with the standard XRD pattern of the standard tetragonal phase BiOBr (JCPDS No. 01-073-2061) of the space group, P4/nmm (129). Its unit cell parameters are a = b = 3.923 Å, c = 8.092 Å. The diffraction peaks of BiOI at 2θ = 10.16°, 30.10°, 32.14°, 37.62°, 45.86°, 51.78°, and 55.56° correspond to {001}, {102}, {110}, {103}, {200}, {114}, and {212} crystal planes of the BiOI crystal, respectively. The position of the BiOI diffraction peak is consistent with the standard XRD pattern of the standard tetragonal phase BiOI (JCPDS No. 01-073-2062). The unit cell parameters are a = b = 3.984 Å, c = 9.128 Å. The diffraction peaks of BiOBr_0.85_I_0.15_ at 2θ = 10.88°, 25.38°, 31.74°, 32.40°, 39.36°, 46.44°, and 57.14° correspond to {001}, {101}, {102}, {110}, {103}, {200}, and {212} crystal planes of the BiOBr_0.85_I_0.15_ crystal, respectively [36].

In BiOBr_0.85_I_0.15_ and Ag/BiOBr_0.85_I_0.15_ composite catalysts with different silver contents, the ratio of I to Br is 0.15:0.85. Therefore, the XRD spectrum of the composite catalyst is closer to that of BiOBr. The diffraction peak of BiOBr_0.85_I_0.15_ is high and sharp, indicating that the samples have good crystallinity. In order to get the lattice parameters, a and c, of the synthesized BiOBr_0.85_I_0.15_ crystal, the Le Bail fitting was used to refine the XRD data (Fullprof, FullProf_Suite Windows (64 bits), Juan RodrÍguez-Carvajal, Laboratory Léon Brillouin (CEA-CNRS), CEA/Saclay, France, https://www.ill.eu/sites/fullprof/php/downloads.html). The lattice parameters, a and c, obtained by fitting are 3.930 Å and 8.270 Å, which is close to the calculated results of the solid solution using Vegard’s Law. Compared with BiOBr_0.85_I_0.15_, the characteristic diffraction peak of Ag/BiOBr_0.85_I_0.15_ has almost no change. Due to the very low concentration and high dispersion of silver in the composite sample, the XRD spectrum shows almost no silver diffraction peak.

### 3.2. Scanning Electron Microscopy (SEM), Ttransmission Electron Microscopy (TEM), and Energy-Dispersive X-ray (EDX)

**Figure 2:**
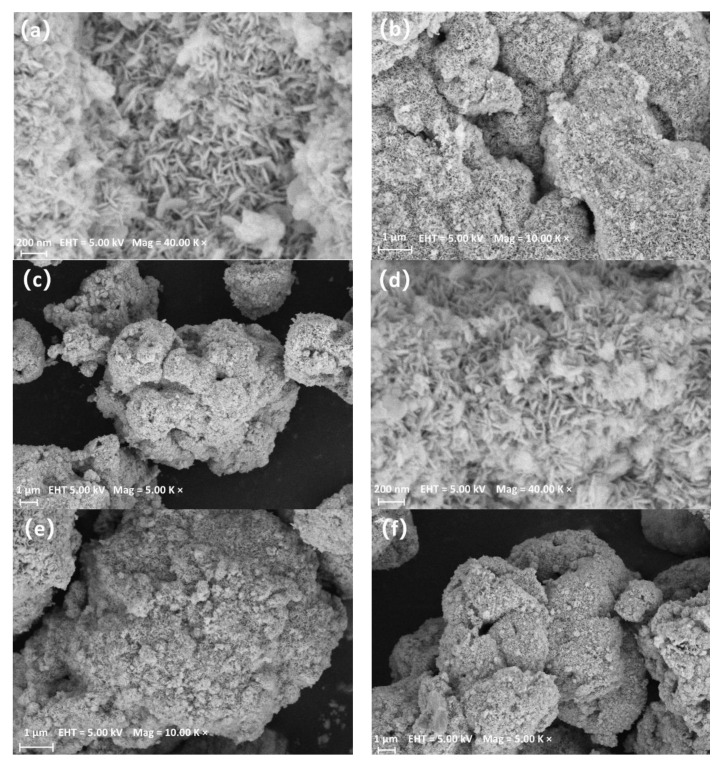
SEM images of BiOBr_0.85_I_0.15_, (**a**–**c**); 1.0% Ag/BiOBr_0.85_I_0.15_, (**d**–**f**).

Because the SEM and TEM images of the as-prepared samples look very similar, only the pictures of the BiOBr_0.85_I_0.15_ and 1.0% Ag/BiOBr_0.85_I_0.15_ are presented. Figure 2a–c show the morphology and microstructure of the BiOBr_0.85_I_0.15_ composite catalyst. It can be seen from Figure 2a that the BiOBr_0.85_I_0.15_ is an irregular lump structure composed of many two-dimensional nanosheets with a width of 0.2~1.0 μm and a thickness of about 10~20 nm. Figure 2c shows that the width of the irregular lump structure is about 4.0~10.0 μm. Figure 2d–f show the morphology and microstructure of 1.0% Ag/BiOBr_0.85_I_0.15_ composite. In terms of morphology, 1.0% Ag/BiOBr_0.85_I_0.15_ has no obvious difference from BiOBr_0.85_I_0.15_, and also presents an irregular lump structure with a porous surface.

**Figure 3:**
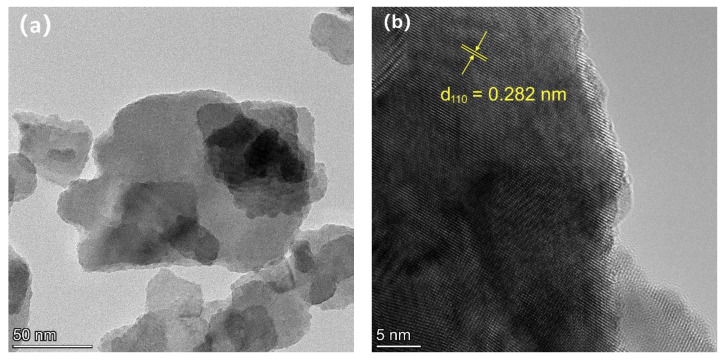

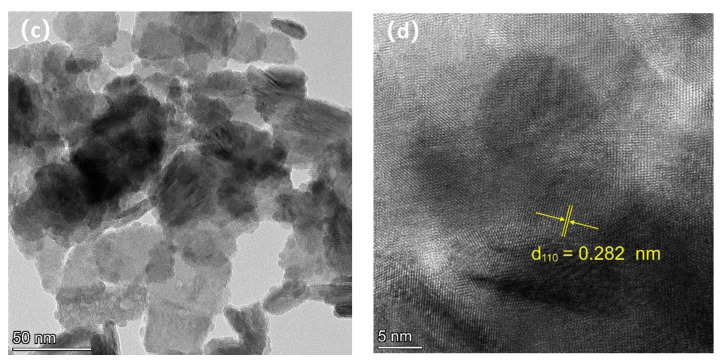
TEM images of BiOBr_0.85_I_0.15_, (**a**,**b**); 1.0% Ag/BiOBr_0.85_I_0.15_, (**c**,**d**).

Figure 3a,b show the TEM and HRTEM images of the prepared BiOBr_0.85_I_0.15_ composite. Figure 3b shows the BiOBr_0.85_I_0.15_ with high crystallinity and clear lattice stripes. The continuous lattice stripes with a crystal plane spacing of 0.282 nm match well with the {110} plane of tetragonal-phase BiOBr (JCPDs No. 01-073-2061). Figure 3c,d show the TEM and HRTEM images of the prepared 1.0% Ag/BiOBr_0.85_I_0.15_ composite. Figure 3d also shows the continuous lattice stripes with a crystal plane spacing of 0.282 nm, which match well with the {110} crystal plane of tetragonal-phase BiOBr [45].

**Figure 4:**
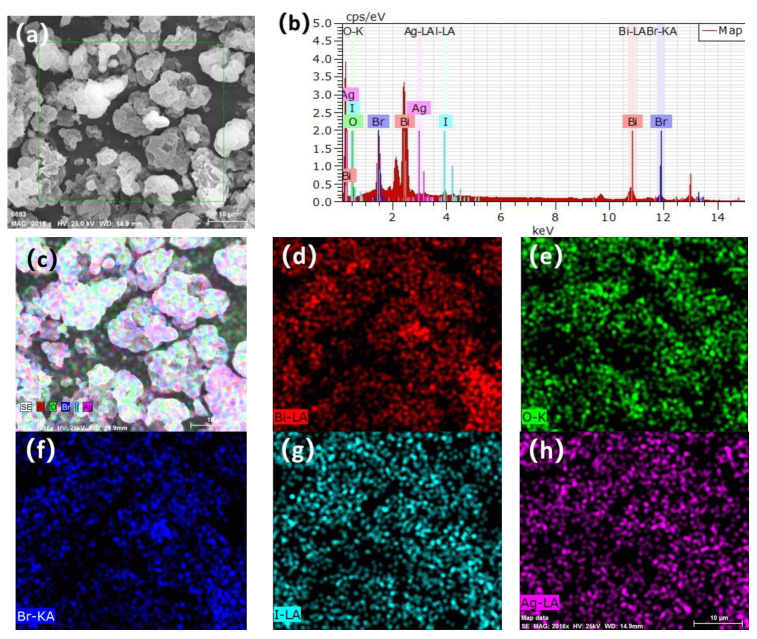
EDS mapping of 1.0% Ag/BiOBr_0.85_I_0.15_: SEM image (**a**), EDS spectrum (**b**), map of Bi, O, Br, I and Ag (**c**), map of Bi (**d**), map of O (**e**), map of Br (**f**), map of I (**g**), map of Ag (**h**).

The elemental composition and distribution of the 1.0% Ag/BiOBr_0.85_I_0.15_ sample are presented in Figure 4. The composite catalyst only contains Bi, O, Br, I, and Ag elements, which is consistent with the theoretical element composition of the composite photocatalyst sample. In addition, the distribution of the above five elements is very uniform, which indicates that silver nanoparticles are uniformly loaded on BiOBr_0.85_I_0.15_ composite nanoparticles. According to the test results of EDX, the rough atomic concentrations of I and Br of the sample surface are 4.76% and 30.18%, respectively. The concentration ratio of I to Br is 15.77:100, which is close to 0.15:0.85 (17.65:100).

### 3.3. X-ray Photoelectron Spectroscopy (XPS)

**Figure 5:**
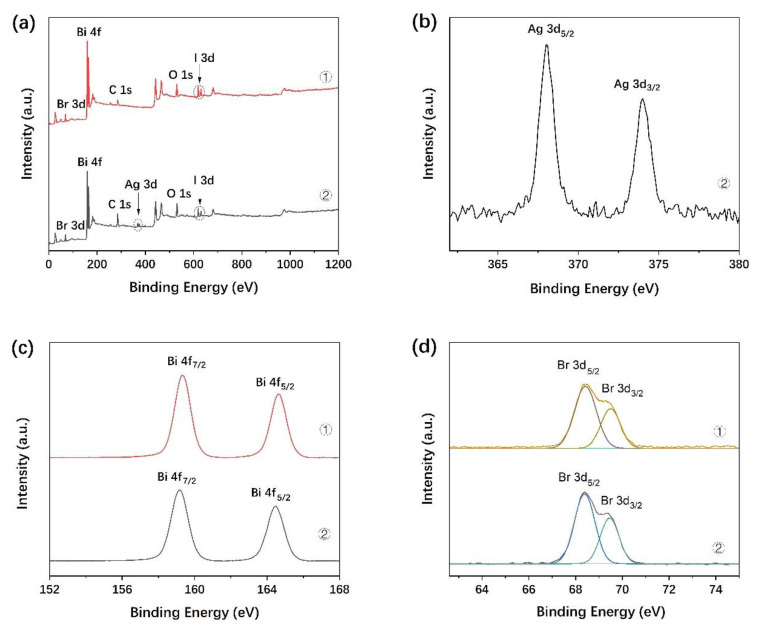

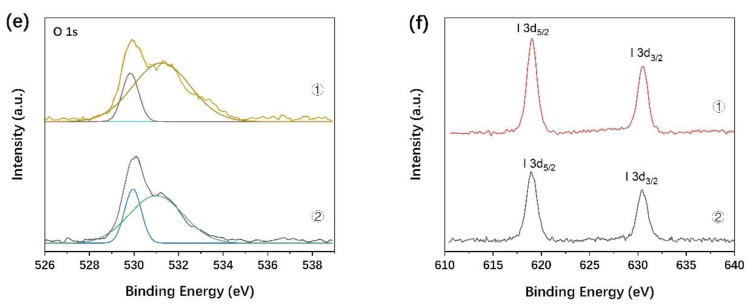
XPS spectra of BiOBr_0.85_I_0.15_ (sample ①) and 1.0% Ag/BiOBr_0.85_I_0.15_ (sample ②): survey spectra (**a**), Ag 3d (**b**), Bi 4f (**c**), Br 3d (**d**), O 1s (**e**), and I 3d (**f**).

The XPS spectra of the BiOBr_0.85_I_0.15_ and 1.0% Ag/BiOBr_0.85_I_0.15_ samples in Figure 5 provide the chemical composition and valence state of the surface elements. All the XPS peak positions are calibrated by C 1s as reference, with a binding energy of 284.8 eV. Figure 5a shows the survey spectra of the BiOBr_0.85_I_0.15_ and 1.0% Ag/BiOBr_0.85_I_0.15_. Only C, O, Bi, Br, and I are detected in BiOBr_0.85_I_0.15_, and C, O, Bi, Br, I, and Ag in 1.0% Ag/BiOBr_0.85_I_0.15_, which indicates the high purity of the samples. The Ag3d_5/2_ and Ag3d_3/2_ spin-orbital splitting photoelectrons for 1.0% Ag/BiOBr_0.85_I_0.15_ in Figure 5b are located at binding energies of 368.0 and 374.0 eV, respectively, which are assigned to metallic-state Ag [46]. As shown in Figure 5c, the two peaks at about 164.5 eV and 159.2 eV are attributed to Bi 4f_5/2_ and Bi 4f_7/2_, respectively, which are characteristic of Bi^3+^ in the BiOX material [47]. The two peaks in Figure 5d can be identified from the Br 3d spectra. The peak of 69.5 eV corresponds to the Br 3d_3/2_, whereas the peak located at 68.4 eV can be attributed to Br 3d_5/2_ in Ag/BiOBr_0.85_I_0.15_ and BiOBr_0.85_I_0.15_, respectively, indicating the presence of Br^−^. Figure 5e shows the high-resolution XPS spectra of the O 1s, which can be fitted into two peaks. The main peaks at about 530.0 eV are attributed to the Bi-O bonds in [Bi_2_O_2_] slabs of the BiOX layered structure, and the small peaks at 531.2 eV are assigned to the hydroxyl groups on the surface in Ag/BiOBr_0.85_I_0.15_ and BiOBr_0.85_I_0.15_ samples, respectively. The two peaks located at 630.5 and 619.0 eV in Figure 5f correspond to I 3d_3/2_ and I 3d_5/2_ in the BiOBr_0.85_I_0.15_ structure, respectively [36]. As a result, the chemical compositions and valence states presented in the XPS spectra are consistent with the composition of the Ag/BiOBr_0.85_I_0.15_ and BiOBr_0.85_I_0.15_.

### 3.4. N_2_ Adsorption–Desorption Isotherms

**Figure 6:**
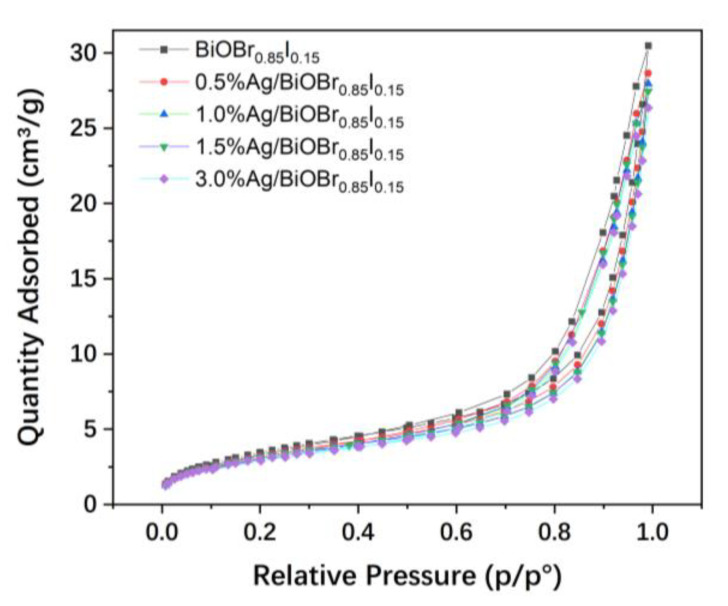
N_2_ adsorption–desorption isotherms.

The N_2_ adsorption–desorption isotherms of the samples are presented in Figure 6. The BET surface areas of the as-prepared samples were tested and calculated to be 13.30, 12.23, 11.89, 11.80, and 11.29 m^2^/g for the BiOBr_0.85_I_0.15_, 0.5% Ag/BiOBr_0.85_I_0.15_, 1.0% Ag/ BiOBr_0.85_I_0.15_, 1.5% Ag/BiOBr_0.85_I_0.15_, and 3.0% Ag/BiOBr_0.85_I_0.15_ samples, respectively. The BET surface areas of Ag/BiOBr_0.85_I_0.15_ samples are a little smaller than that of the BiOBr_0.85_I_0.15_ sample. With the increase of silver loading, the specific surface areas of the samples decreased slightly.

### 3.5. UV-Visible Diffuse Reflectance Spectra (UV-Vis DRS)

**Figure 7:**
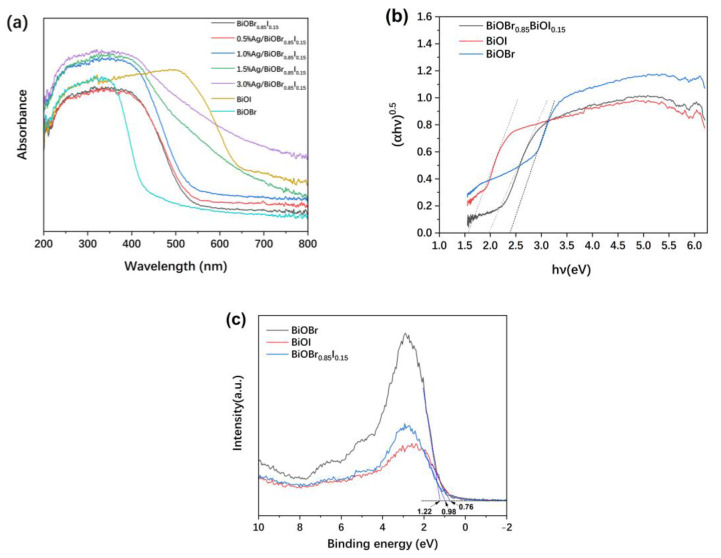
UV-vis diffuse reflectance spectra (**a**); the bandgap value, estimated by a related curve of (αh*ν*)^0.5^ versus photon energy (**b**); and the VB-XPS spectra of BiOBr, BiOI, and BiOBr_0.85_I_0.15_ (**c**).

The UV-visible diffuse reflectance spectra of the samples in the range of 200~800 nm are shown in Figure 7a. The BiOBr_0.85_I_0.15_ sample has a stronger absorption of UV light than that of visible light. In the visible region, the light absorption capacity of the sample decreases significantly with the increase of wavelength. With the increase of the silver loading, the absorption of light, especially visible light by the Ag/BiOBr_0.85_I_0.15_ composite, increases significantly. In fact, the as-prepared BiOBr_0.85_I_0.15_ is orange-yellow. Silver nanoparticles are black, which is due to the absorption of visible light caused by surface plasmon resonance. The color of the Ag/BiOBr_0.85_I_0.15_ catalysts become darker with the increase of silver loading.

The bandgap value can be estimated by a related curve of (αh*ν*)^0.5^ versus photon energy (h*ν*) according to the Kubelka–Munk function, αh*ν* = A(h*ν* − E_g_)^n/2^, where α, h, *ν*, and A stand for absorption coefficient, Planck constant, light frequency, and proportionality, respectively [48]. The value of n is determined by the optical transition mode of the semiconductor. For BiOBr and BiOI, the value of n is 4 [36].

It can be seen from Figure 7b that the band gap values of BiOBr and BiOI are estimated to be 2.39 eV and 1.58 eV, respectively, and the band gap of the BiOBr_0.85_I_0.15_ composite is 1.98 eV. The band gap energy of the composite is between the energy of BiOBr and BiOI, and is obviously smaller than that of BiOBr, which enhances its absorption of visible light, and is conducive to the photocatalytic reaction.

As shown in Figure 7c, the valence band X-ray photoelectron spectra (VB-XPS) of the samples were investigated. The valence band potential of BiOBr, BiOI, and BiOBr_0.85_I_0.15_ were found to be at 1.22, 0.76, and 0.98 eV, respectively. According to the equation of E_CB_ = E_g_ − E_VB_ [49], the conduction band (CB) edges of BiOBr, BiOI, and BiOBr_0.85_I_0.15_ are calculated to be −1.17, −0.82, and −1.00 eV, respectively; this indicates that BiOBr_0.85_I_0.15_ has elevated oxidizing ability compared to BiOI, through a higher VB position.

### 3.6. Electron Spin Resonance (ESR) Spectroscopy

**Figure 8:**
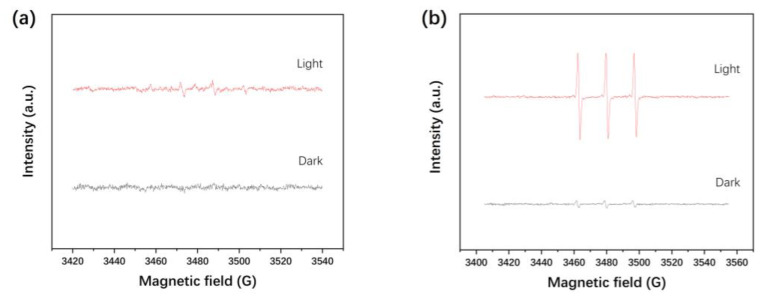

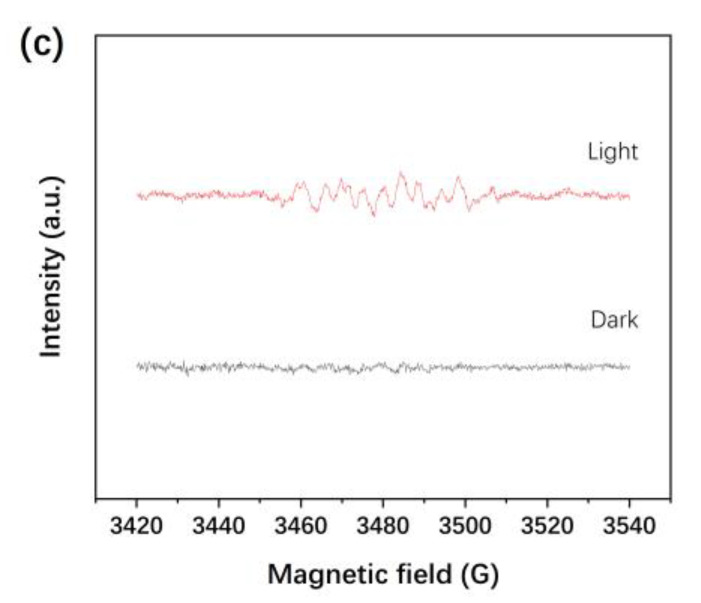
The ESR spetra of DMPO-·OH (**a**), the ESR spetra of TEMP-^1^O_2_ (**b**), and the ESR spetra of DMPO-·O_2^−^_ (**c**) (the sample is 1.0% Ag/BiOBr_0.85_I_0.15_ irradiated under the xenon lamp light for 80 s).

ESR technology is used to detect reactive oxygen species (·OH, ^1^O_2_, and ·O_2^−^_) during the photodegradation process. Figure 8a–c show the ESR signals of DMPO-·OH, TEMP-^1^O_2_, and DMPO-·O_2^−^_ adduct with or without light irradiation, respectively. The signals of the reactive oxygen species are almost undetectable or not obvious in the dark. Under visible light irradiation, the six characteristic peaks of DMPO-·O_2^−^_ and three characteristic peaks of TEMP-^1^O_2_ are very strong, but the four characteristic peaks of ·OH are not significant [36,50]. OH, ^1^O_2_, and ·O_2^−^_ are detected in the visible photocatalytic degradation system, which confirmed that these free radicals are produced in the photocatalytic process over Ag/BiOBr_0.85_I_0.15_ nanoparticles. Moreover, ^1^O_2_ and ·O_2^−^_ are the main oxidation species, whereas ·OH is not important under the reaction condition.

In photocatalysis, holes can oxidize H_2_O or OH^−^ to generate ·OH, whereas ·O_2^−^_ is mainly generated by O_2_ capturing *e*^−^. The generation of ·OH depends on whether the E_VB_ is higher than the redox potential of ·OH/OH^−^ (1.99 eV), and the generation of ·O_2^−^_ depends on whether the E_CB_ of the semiconductor is lower than the redox potential of ·O_2^−^_/O_2_ (−0.33 eV) [36]. As is stated above, the E_VB_ and E_CB_ of BiOBr_0.85_I_0.15_ are calculated to be 0.98 eV and −1.00 eV, respectively. Therefore, in the photocatalyst surface, ·O_2^−^_ can be directly produced, but it is difficult to directly produce ·OH. However, ·OH can be generated by further reduction of ·O_2^−^_, which is an indirect way to form ·OH. The ^1^O_2_ is likely to be formed by the oxidation of ·O_2^−^_, which is generated by the reduction of surface-adsorbed O_2_ [51]. Moreover, semiconductor catalysts can produce photogenerated electron–hole pairs (excitons, *e*^−^ − h^+^) under UV or visible light radiation. The electrostatic Coulomb interaction of photogenerated exciton usually produces a strong exciton effect, and the accompanying energy transfer can excite O_2_ to produce ^1^O_2_ [52]. The relevant reactions are as follows.

*e*^−^ + O_2_ ⟶ ·O_2^−^_ (1)

·O_2^−^_ + *e*^−^ + 2H^+^ ⟶ H_2_O_2_ (2)

·O_2^−^_ + H_2_O ⟶ ·OH + OH^−^ +O_2_ (3)

·O_2^−^_ + h^+^ ⟶ ^1^O_2_ (4)

Catalyst + h*ν* ⟶ Catalyst* (5)

Catalyst* + ^3^O_2_ ⟶ Catalyst + ^1^O_2_ (6)

### 3.7. Photocatalytic Degradation of Ammonia Nitrogen

**Figure 9:**
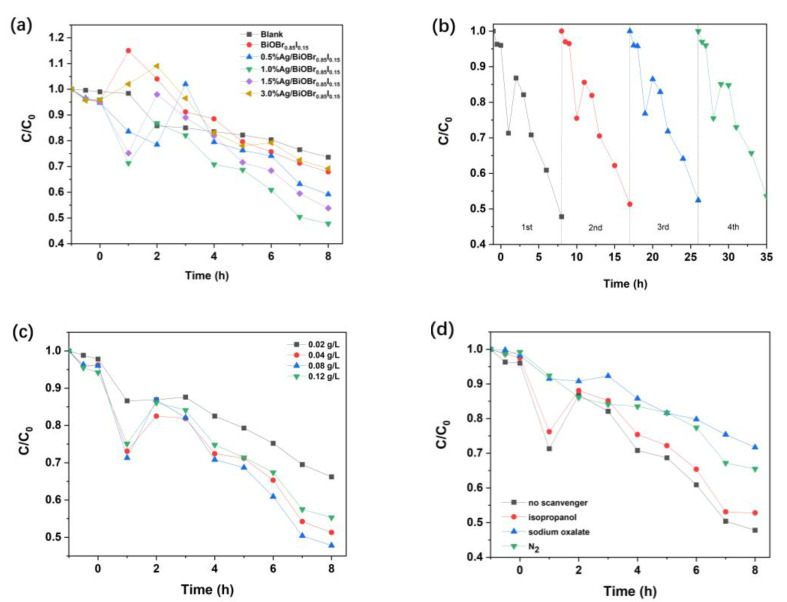
Photocatalytic activities of different catalysts (**a**), cycling runs of 1.0% Ag/BiOBr_0.85_I_0.15_ sample for the removal of NH_4_^+^-N (**b**), effect of catalyst dosage on NH_4_^+^-N degradation rate (**c**), and the NH_4_^+^-N degradation efficiencies of 1.0% Ag/BiOBr_0.85_I_0.15_ under different scavengers (**d**).

Figure 9 shows the ability of the as-prepared catalysts to degrade NH_4_^+^-N in the water sample from the recirculating aquaculture system. The photocatalytic capability comparison between BiOBr_0.85_I_0.15_ and Ag/BiOBr_0.85_I_0.15_ samples with different Ag loading is shown in Figure 9a. Under the blank condition, the NH_4_^+^-N value decreased slowly to 73.6% in 8 h under the light irradiation, which implied that the light radiation alone could degrade about 26% of NH_4_^+^-N in the water sample. In the presence of BiOBr_0.85_I_0.15_, about 32% NH_4_^+^-N was degraded within 8 h. Compared with the degradation result of the blank experiment, the addition of BiOBr_0.85_I_0.15_ did not obviously improve the degradation efficiency of NH_4_^+^-N. The degradation rates of NH_4_^+^-N in the presence of the photocatalysts, 0.5% Ag/ BiOBr_0.85_I_0.15_, 1.0% Ag/ BiOBr_0.85_I_0.15_, and 1.5% Ag/BiOBr_0.85_I_0.15_, were 40.8%, 52.2%, and 46.2%, respectively. However, the catalytic capacity of the photocatalyst did not continue to increase with the increase of silver loading. The degradation rate of NH_4_^+^-N with 3.0% Ag/ BiOBr_0.85_I_0.15_ was 30.8%, which is significantly lower than that of 1.0% Ag/BiOBr_0.85_I_0.15_. The 1.0% Ag/BiOBr_0.85_I_0.15_ showed the best photocatalytic ability.

Ag nanoparticles on the surface of semiconductors can become electron capture centers, which can improve the separation efficiency of carriers, effectively inhibit the recombination of photogenerated electrons–holes, thus accelerating the photocatalytic reaction rate and improving the photocatalytic activity of Ag/BiOBr_0.85_I_0.15_ nanoparticles. However, Ag nanoparticles may become the recombination center of carriers when the Ag loading is further increased. On the other hand, too many Ag nanoparticles covering the surface of the catalyst will also affect the effective absorption of light by the catalyst [53].

**Figure 10:**
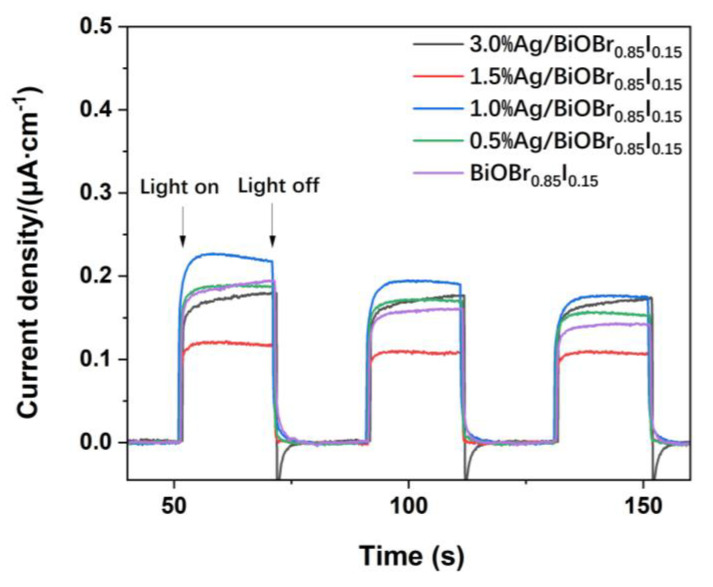
Transient photocurrent response for BiOBr_0.85_I_0.15_ and Ag/BiOBr_0.85_I_0.15_ photocatalysts.

Figure 10 shows that the 1.0% Ag/BiOBr_0.85_I_0.15_ nanocomposite displays the highest photocurrent response. It proves that the loading of Ag nanoparticles could enhance the separation efficiency of photogenerated charges of the catalyst. The photocurrent of the 0.5% Ag/BiOBr_0.85_I_0.15_ sample is close to or slightly higher than that of BiOBr_0.85_I_0.15_. The photocurrent of 1.5% Ag/BiOBr_0.85_I_0.15_ is significantly lower than that of BiOBr_0.85_I_0.15_. The results are basically in line with the actual degradation performance of the catalysts. However, it is worth noting that the photocurrent of 3.0% Ag/BiOBr_0.85_I_0.15_ is higher than that of 1.5% Ag/BiOBr_0.85_I_0.15_, and also higher than that of BiOBr_0.85_I_0.15_ samples. This may be due to the significant increase of the photocurrent generated by the surface plasmon resonance of silver nanoparticles. The surface plasmon resonance photocurrent increases significantly with the increase of silver loading. However, since the oxidation of photogenerated holes on the surface of silver nanoparticles is not strong, although the actual photocurrent increases, it cannot effectively promote the degradation performance of the composite catalyst [54,55].

The results shown in Figure 9a indicate that the photocatalysts with different silver loading have different removal effects on NH_4_^+^-N. It is worth noting that the degradation curves of the experiments with catalysts show significant fluctuation within 8 h, especially a remarkable rise in the initial 1~3 h, whereas the whole degradation curve of the blank experiment shows a downward trend. The fluctuation of the degradation curve implies the complexity of the photodegradation process. The significant increase of the NH_4_^+^-N value in the degradation process may be related to the photocatalytic transformation of nitrogen-containing compounds. There are many complex nitrogen-containing organic compounds in the water of the recirculating aquaculture system, which mainly come from the feed, excreta, and secretions of fish. The existence of the photocatalyst makes the reaction system produce more active oxygen radicals and holes (h^+^), which leads to a series of subsequent reactions to convert organic nitrogen into NH_4_^+^-N. With the transformation of nitrogen-containing organic matter into NH_4_^+^-N under the photocatalytic condition, the NH_4_^+^-N value increased in the first a few hours, and then began to decline in a fluctuating way, and was, finally, significantly lower than the initial value [56]. Under the same photoreaction condition, different photocatalysts have different conversion rates of nitrogen-containing organic compounds and degradation rates of NH_4_^+^-N, so the fluctuation of their degradation curves is not completely synchronous.

Figure 9b shows the recycling stability of 1.0% Ag/BiOBr_0.85_I_0.15_ used for removing NH_4_^+^-N. It indicates that the 1.0% Ag/BiOBr_0.85_I_0.15_ sample kept stable photocatalytic activity after four-times cycling usage under the simulated sunlight radiation.

The effect of catalyst dosage on the NH_4_^+^-N degradation rate is shown in Figure 9c. The removing rates of NH_4_^+^-N are 33.8%, 48.7%, 52.2%, and 44.7%, respectively, when the photocatalyst dosage increased from 0.02 g/L to 0.12 g/L. With the increase of catalyst dosage, the final degradation rate of NH_4_^+^-N in 8 h increased first, and then decreased. This may be because when the separation efficiency of the photogenerated electron–hole pairs is certain, the greater the amount of the photocatalyst, and the greater the number of surface active sites involved in the catalytic reaction, so as to improve the degradation rate of NH_4_^+^-N. However, when the dosage increased to a certain amount, the removing rate of NH_4_^+^-N decreased. This may be due to the serious agglomeration of photocatalytic materials due to excessive dosage, resulting in the reduction of the surface area effectively used for the catalytic reaction. Moreover, it will also make the solution turbid, and the suspended particles in the solution will scatter the light to prevent the catalyst from making full use of light and to reduce the photocatalytic efficiency.

In order to investigate the main active species in the photocatalytic degradation of NH_4_^+^-N in recirculating aquaculture water under simulated sunlight, active species scavengers, sodium oxalate, isopropanol, and nitrogen (N_2_), were added to consume or reduce the active species hole (h^+^), ·OH, ·O_2^−^_, and ^1^O_2_, respectively [36,57]. The concentration of ammonium oxalate and isopropanol is about 5 mM. In the beginning of degradation, the water sample was flushed with nitrogen to remove most of the oxygen. The effect of scavengers on NH_4_^+^-N degradation is presented in Figure 9d. The degradation rate of NH_4_^+^-N decreased from 52.2% to 41.2%, 34.5%, and 28.3% after aerating N_2_ or adding isopropanol and sodium oxalate, respectively, which indicated the degradation efficiency was obviously inhibited. The results suggest that in this degradation system, the important oxidation species are h^+^, ·O_2^−^_, and ^1^O_2_. ·OH radicals make a minor contribution to NH_4_^+^-N degradation. This is consistent with the ESR characterization results.

## 4. Conclusions

In this work, a series of Ag/BiOBr_0.85_I_0.15_ photocatalysts with different Ag loading (0.5, 1.0, 1.5, and 3.0% mol%) were successfully synthesized through a solvothermal and photoreduction method. The as-prepared photocatalysts were applied to the photocatalytic degradation of NH_4_^+^-N in circulating aquaculture water. The influence of Ag loading on the photocatalytic activity of Ag/BiOBr_0.85_I_0.15_ photocatalysts was investigated. The ESR detection and free radical scavenging experiment results suggest that h^+^, ^1^O_2_, and ·O_2^−^_ are the main oxidation species in the photocatalytic reaction system. The degradation results showed that the appropriate amount of silver loading (0.5, 1.0, and 1.5 mol%) could improve the catalytic activity of the catalyst, but a further increase of silver loading will inhibit the photocatalytic degradation ability of the catalyst. Among the synthesized photocatalysts, the 1.0% Ag/BiOBr_0.85_I_0.15_ sample showed the highest photoactivity for the NH_4_^+^-N degradation; this may be due to the inhibition of the recombination of photogenerated electrons–holes. The 1.0% Ag/BiOBr_0.85_I_0.15_ sample also displayed stable photocatalytic activity after four-times cycling usage under the simulated sunlight radiation. Therefore, 1.0% Ag/BiOBr_0.85_I_0.15_ nanocomposites are good candidates for applications as sunlight photocatalysts.

## Data Availability

The data presented in this study are available on request from the corresponding author or the first author.
